# Brief Exercise Counseling and High-Intensity Interval Training on Physical Activity Adherence and Cardiometabolic Health in Individuals at Risk of Type 2 Diabetes: Protocol for a Randomized Controlled Trial

**DOI:** 10.2196/11226

**Published:** 2019-03-26

**Authors:** Jessica E Bourne, Jonathan P Little, Mark R Beauchamp, Julianne Barry, Joel Singer, Mary E Jung

**Affiliations:** 1 Centre for Exercise, Nutrition and Health Sciences School of Policy Studies University of Bristol Bristol United Kingdom; 2 School of Health and Exercise Sciences University of British Columbia Kelowna, BC Canada; 3 School of Kinesiology University of British Columbia Vancouver, BC Canada; 4 School of Population and Public Health University of British Columbia Vancouver, BC Canada

**Keywords:** exercise, type 2 diabetes, high-intensity interval training, prediabetes

## Abstract

**Background:**

Worldwide incidence of type 2 diabetes (T2D) is rapidly increasing. Given the numerous negative health consequences associated with T2D, prevention of this disease has become a priority. Lifestyle changes, including regular exercise, can reduce the onset of T2D in those at elevated risk. However, long-term adherence to exercise is often poor in this population. Existing lifestyle interventions targeting exercise are labor intensive and costly for staff and participants. Evidence-informed counseling delivered in a manner that reduces dependence on staff and facilitates self-regulatory skills could alleviate time and financial barriers while promoting independent exercise.

**Objective:**

This protocol outlines the design, recruitment, and proposed analysis of a brief, 2-week evidence-informed exercise counseling intervention combined with either high-intensity interval training (HIIT) or traditional moderate-intensity continuous training (MICT).

**Methods:**

Small Steps for Big Changes is a 2-arm randomized controlled trial that will examine the effectiveness of combining brief exercise counseling with HIIT or MICT on adherence to moderate and vigorous exercise over 1 year. Cardiorespiratory fitness will be assessed at baseline, post intervention (2 weeks), and at 6- and 12-month follow-up. Physical activity behavior will be examined at baseline, post intervention, and 3-, 6-, 9-, and 12-month follow-up. The impact of the intervention on psychosocial outcomes pertinent to exercise adherence will be examined.

**Results:**

Data collection was complete in March 2017. Data analysis is currently underway, and the first results are expected to be submitted for publication in 2019.

**Conclusions:**

The results of this brief intervention have the potential to inform future public health efforts designed to increase exercise in individuals at risk of T2D.

**Trial Registration:**

ClinicalTrials.gov NCT02164474; https://clinicaltrials.gov/ct2/show/NCT02164474 (Archived by WebCite at http://www.webcitation.org/74Hx1ipj6)

**International Registered Report Identifier (IRRID):**

DERR1-10.2196/11226

## Introduction

### Background

Type 2 diabetes (T2D) is one of the fastest growing diseases in Canada, with approximately 60,000 new cases diagnosed each year [1]. Globally, it is estimated that 592 million individuals will be diagnosed with T2D by 2035 [2]. Given the numerous negative health outcomes associated with T2D, including heart disease, retinopathy, cataracts, neuropathies, and kidney failure [3-6], increasing attention has turned to intervening with individuals at increased risk of developing T2D (ie, individuals living with *prediabetes*).

Physical activity has been highlighted as beneficial for preventing the progression of prediabetes to T2D [7,8]. However, individuals at risk for T2D often fail to meet the physical activity guidelines of 150 minutes of moderate-to-vigorous aerobic exercise per week [9,10]. Innovative trials, such as the Diabetes Prevention Program (DPP), have demonstrated that a lifestyle intervention incorporating 150 minutes of moderate-intensity physical activity per week can reduce the progression of prediabetes to T2D by up to 58% compared with a non-exercise control and is almost twice as effective as the leading pharmaceutical intervention [11]. At 7-year follow-up, moderate-to-vigorous physical activity behavior was higher among individuals that completed the Diabetes Prevention Program Outcome Study (DPPOS) compared with an equivalent cohort [12]. The DPP and DPPOS follow-up represented a landmark trial designed to assess the potency of lifestyle modification for the prevention of T2D. The trial involved extensive patient contact and monetary incentives to ensure adherence to the prescribed 150 minutes of exercise per week and dietary and weight loss goals. The cumulative cost of the DPP and DPPOS for the lifestyle intervention group was US $4601 per person [13]. Community sites aspiring to achieve similar rates of adherence to physical activity are likely to be constrained by financial and time burdens associated with implementing (and participating in) the DPP. The intensity and expense associated with the DPP and DPPOS could limit the scalable implementation of such interventions. There is an urgent need for affordable and practical interventions that can be delivered in real-world health care or community settings with similar degrees of effectiveness [14]. *Small Steps for Big Changes* is a brief, evidence-informed lifestyle intervention that seeks to overcome the limitations of feasibility and sustainability in previous interventions with the aim of increasing physical activity adherence in individuals at elevated risk of T2D.

### Promoting Physical Activity Adherence

To increase the potential for long-term behavior change, it is important to target theoretically proposed mechanisms of change and incorporate evidence-based behavior change strategies to maximize potential intervention success. Hardeman et al [15] refer to this as a causal modeling approach to intervention development. Interventions that incorporate psychological theory and empirical evidence about how best to support behavior change have been found to be effective [16,17].

Social cognitive theory (SCT) provides a conceptual framework through which to design physical activity interventions. A key construct of SCT is the confidence in one’s ability to carry out and self-manage behavior (ie, *self-regulatory efficacy beliefs*). Self-regulatory efficacy is essential in promoting long-term engagement in behaviors such as physical activity [18]. Another form of self-efficacy, concurrent self-regulatory efficacy, reflects an individual’s confidence to manage health and life goals simultaneously and has been shown to predict physical activity levels [19]. Self-efficacy has been consistently found to be a significant predictor of the adoption and maintenance of physical activity behavior [20,21] and has been reported as the most influential construct on physical activity behavior change [22]. In relation to T2D, self-efficacy has been shown to increase after free-living exercise interventions [23] and has been found to mediate the relationship between intervention delivery and objectively measured physical activity behavior [24]. In addition, personally held efficacy beliefs influence the outcomes an individual anticipates attaining by engaging in the target behavior [25]. These outcome expectations and the value individuals attach to these outcomes can impact physical activity motivation. Individuals who perceive little value or likelihood of achieving benefits associated with physical activity are less likely to self-manage their behavior [25]. As such, fostering positive beliefs about the outcomes an individual will stand to gain from physical activity, which are of value to the individual, will positively influence motivation.

Given the role of self-efficacy beliefs and outcomes expectations as psychological mechanisms of behavior change, a growing body of research has sought to examine the utility and effects of different behavior change techniques (BCTs) in relation to both social cognitions and thereafter physical activity behavior in individuals at risk of T2D [26-28]. For example, Williams and French [27] reported that action planning, reinforcing effort or progress toward behavior, and providing instruction produced significantly higher self-efficacy and physical activity effect sizes compared with interventions that did not incorporate these techniques. A meta-regression by Michie et al [28] reported that interventions that incorporated self-monitoring and self-regulation techniques were significantly more effective at increasing general physical activity behavior than those that did not include these techniques. In the context of T2D, Cradock et al [29] highlighted 4 BCTs associated with reductions in glycated hemoglobin. These techniques included instruction on how to perform a behavior, behavioral practice rehearsal, demonstration of the behavior, and action planning.

*Small Steps for Big Changes* was developed to target the underlying theoretical- and evidence-informed mechanisms of behavior change within a brief intervention to promote long-term physical activity behavior in individuals at risk of T2D. Findings from a pilot feasibility study provided preliminary evidence that the *Small Steps for Big Changes* intervention protocol resulted in significant increases in self-regulatory efficacy and outcome expectations and a significant increase in purposeful moderate-to-vigorous physical activity (MVPA) 1 [30] and 6 months following the program [31].

### Moderate-Intensity Continuous Training: A Traditional Approach

In addition to consideration of the psychological constructs to be targeted within a physical activity intervention, it is also important to consider the modality of exercise prescribed. Traditionally, individuals at risk of T2D embarking on a new exercise regime are prescribed moderate-intensity continuous training (MICT; eg, steady-state walking), with limited free-living adherence typically reported [11]. Given the low rates of adherence to this exercise prescription [32], alternative training protocols, such as high-intensity interval training (HIIT), are increasingly being explored.

### High-Intensity Interval Training: A New Approach for Individuals With Prediabetes

HIIT has received considerable attention as it elicits positive metabolic and cardiovascular adaptations that are similar, or even superior, to MICT in a variety of populations with lower time commitments [33,34]. Specific to diabetes prevention, a recent meta-analysis demonstrated that HIIT leads to greater improvements in insulin resistance compared with MICT [35]. Given the positive health adaptations, HIIT may represent a promising physical activity strategy for individuals with prediabetes.

HIIT involves brief, repeated bursts of vigorous exercise separated by periods of recovery. In a cross-over study of inactive adults, comparable exercise enjoyment and confidence were found between HIIT and MICT, with a greater proportion of participants reporting a preference to engage in HIIT (62%) over MICT (20%) after a single bout of each exercise modality [36]. Similar results are reported in individuals with overweight and obesity [37-39]. Surprisingly, few studies have examined free-living adherence to HIIT in individuals at risk of T2D, despite the known benefits of HIIT on glucose control in this population [40]. In our previous pilot study of individuals living with prediabetes, participants demonstrated greater free-living adherence to HIIT when compared with MICT 1 month following 10 sessions of supervised training and counseling [30]. In addition, accelerometer analysis of physical activity behavior revealed that participants randomized to the HIIT protocol engaged in significantly more vigorous exercise than those randomized to the MICT protocol. Furthermore, after 6 months of independent exercise, participants in the HIIT condition engaged in significantly more moderate-intensity activity compared with those randomized to MICT [31]. Although these initial findings are promising, further research is needed to confirm these results and determine whether individuals with prediabetes can adhere to HIIT over the *long term* (ie, 12 months). Furthermore, trials with greater power are needed to explore whether psychological responses associated with these modalities of activity impact adherence rates.

### Small Steps for Big Changes Objectives

The objectives of this randomized controlled trial are to:

1. Compare differences in cardiorespiratory fitness between HIIT and MICT at 6 and 12 months after the intervention.
2. Examine accelerometry-measured purposeful moderate-to-vigorous physical activity in bouts of ≥10 minutes (MVPA10+) and total accelerometer counts throughout the follow-up year (ie, 3, 6, 9, and 12 months) after the HIIT and MICT intervention. Self-reported adherence to HIIT and MICT exercise prescriptions will be collected at 3-, 6-, 9-, and 12-month follow-up.
3. Examine the following psychological outcomes to explore their potential relationship with free-living adherence: task self-efficacy, self-regulatory efficacy, concurrent self-regulatory efficacy, self-monitoring, outcome expectations, exercise enjoyment, and instrumental and affective attitudes. In addition, differences in acute affect, in-task enjoyment, and perceived exertion between HIIT and MICT will be compared.
4. Examine differences in cardiometabolic health markers. Glucose control and insulin sensitivity will be assessed at baseline, post intervention, and at 6- and 12-month follow-up. Body composition will be assessed at baseline, post intervention, and at 12-month follow-up. Differences in average blood glucose levels between conditions will be examined immediately after the intervention in a subsample of participants.

## Methods

### Study Design

**Figure 1:**
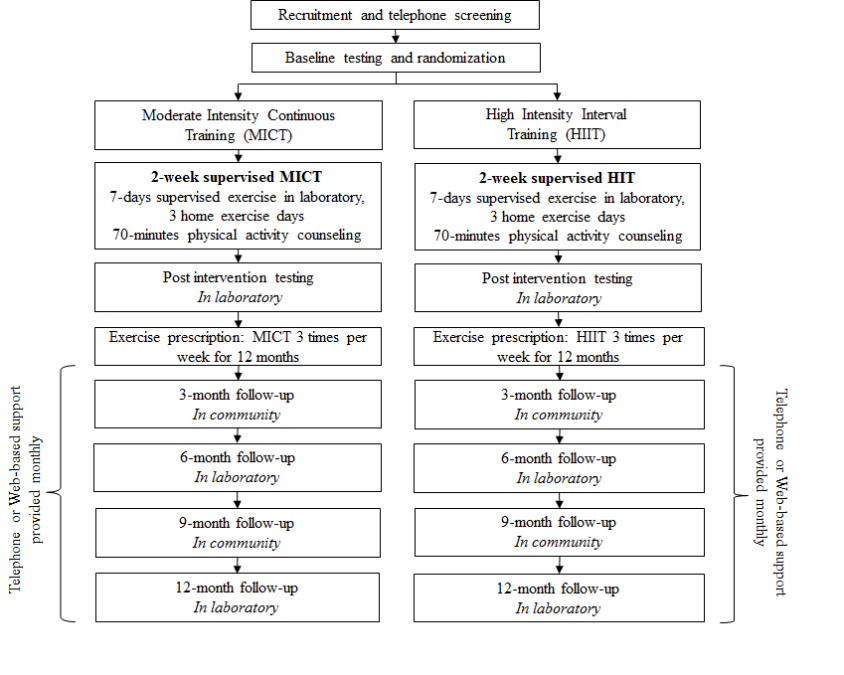
Flow of participants through the Small Steps for Big Changes program.

*Small Steps for Big Changes* is a 2-arm, parallel group randomized controlled trial comparing adherence to HIIT with MICT in individuals at risk of T2D. This single-center trial will be conducted at The University of British Columbia, Okanagan Campus. The design of the study and flow of participants is described in Figure 1. Participants in both conditions will receive a 2-week supervised physical activity intervention consisting of engagement in their randomized exercise modality and brief, targeted counseling. The physical activity counseling will be identical for both conditions. Follow-up assessments will occur at 3, 6, 9, and 12 months. The trial will aim to recruit 100 participants. The supervised training will be conducted in waves (10 waves with approximately 10 participants per wave). All participants will begin supervised training on a Monday. Reporting of this trial protocol follows the Standard Protocol Items: Recommendations for Interventional Trials statement. Future reporting of the results of this trial will follow the Consolidated Standards of Reporting Trials guidelines [41].

### Participants

The study sample will consist of adults aged between 30 years and 65 years; who are physically inactive (ie, engage in 2 or less bouts of moderate and/or vigorous aerobic exercise per week in the last 6 months, assessed using the Godin Leisure Time Physical Activity questionnaire [42]); have a body mass index (BMI) between 25 kg/m^2^ and 40 kg/m^2^; and are cleared to engage in vigorous exercise (via the Physical Activity Readiness Questionnaire for Everyone [PAR-Q+] or further clearance by physician, if needed [43]).

### Recruitment and Screening

Participants will be recruited using posters administered in the local community, social media, newspaper advertisements, and word of mouth. Advertisements will invite inactive adults aged between 30 years and 65 years to contact the researchers about potential participation in a lifestyle change program, specifically designed to assist individuals who wish to become regular exercisers. A contact phone number and email address will be provided for individuals to contact the research team. Individuals who contact the research team will be able to ask questions regarding the program, and if wishing to proceed, will be scheduled for a previsit telephone screening interview. Telephone screening will take approximately 30 minutes during which the research coordinator will outline the time commitment of the study; record the participants’ age, current medications, height, and weight (to calculate BMI); and administer verbally and record responses to the Godin Leisure Time Physical Activity questionnaire [42] and the PAR-Q+ [43]. Following completion of the initial screening questions, the research coordinator will inform the individual that he or she is (1) ineligible to participate in the program with reasons provided; (2) may be eligible, however, based on the results of the PAR-Q+ clearance and completion of the Physical Activity Readiness Medical Examination (ePARmed-X+) [43] will be required from the individual’s family physician; or (3) may be eligible for the program and will be invited to the laboratory for final screening. Participants required to obtain medical clearance will be sent the ePARmed-X+ (electronically or via mail) and must have the form completed and signed by their physician. Potentially eligible individuals will be sent an information sheet containing study details and will be informed that final eligibility status will be shared following completion of a baseline assessment to confirm objective height, weight, and activity status.

### Baseline Testing and Randomization

Baseline testing will last approximately 150 minutes. Participants will be provided with a detailed consent form and will be invited to ask any questions. After written consent is obtained, weight, height, and blood pressure will be taken and a 7-day physical activity recall interview [44] will be conducted to confirm eligibility.

Eligible individuals will be stratified based on sex and then randomly assigned to either the HIIT or MICT exercise conditions. An external statistician, who has no other involvement in the trial, will generate random allocation sequences, which will be linked to an interactive Web page. Permuted blocks of random size will be used. Once an individual is identified as eligible, the research coordinator will access the password-protected Web page, and after entering requisite information on patient eligibility and sex, participants will be randomly allocated. Participants and researchers will be aware of group allocation. Following group assignment, participants will have a fasting blood sample taken, consume a 75 g glucose drink as part of a modified oral glucose tolerance test, complete several Web-based questionnaires, have a second blood sample taken 30 minutes following the glucose drink consumption, undergo a body composition scan, and complete a maximal cardiorespiratory fitness test to exhaustion on a cycle ergometer. At the end of the session, participants will be provided with an accelerometer to wear for 7 consecutive days, commencing the day after baseline testing. Participants will be shown how to wear the device and will be instructed to remove the device while sleeping or for water-based activities. In addition, instruction will be provided to remind participants how to correctly wear the accelerometer. Short message service (SMS) text messages or emails will be sent on days 1, 3, and 5 of monitoring to remind participants to wear the device (see Multimedia Appendix 1 for study measures).

A voluntary subsample of 8 to 10 participants per group will return to the laboratory 3 days after the preassessment for insertion of a continuous glucose monitor (CGM). The CGM will be worn for 2 consecutive days, and participants will be provided with a standardized diet. Participants will return after 2 days of wearing the CGM to have the CGM removed. These 2 additional visits to the laboratory will last approximately 30 minutes in total. No sample size calculation will be conducted as this aspect of the study represents an exploratory aim to determine if CGM can be used to detect changes in glucose control in this population.

### Supervised Exercise Training

In this study, 10 days after completing baseline testing, eligible participants will begin the 2-week supervised exercise training. Participants in each condition will participate in 10 sessions of exercise performed over a 12-day period (ie, Monday to Friday over 2 weeks with Saturday and Sunday as rest days). Seven of these sessions will be supervised in the laboratory and will last approximately 30 to 60 minutes each, while 3 sessions will be conducted at home to foster independence and work through plausible barriers participants may encounter in the future. Each supervised exercise session will occur one-on-one with a counselor and will consist of an exercise component (ie, HIIT or MICT) and 10 minutes of counseling. Participants will be exposed to a variety of exercise formats within their given modality (ie, stationary cycling, treadmill walking, elliptical machine, and outside walking). For 4 of the 7 supervised sessions, participants will be able to self-select the exercise format to encourage autonomy. A total of 2 sessions will be performed on the cycle ergometer during which in-task affect and enjoyment will be assessed to more precisely control exercise intensity and control for the influence of exercise format on enjoyment. The exercise prescriptions for each condition will be progressive in nature (see Table 1) and matched for estimated external work.

**Table 1:** Two-week intervention exercise prescription.

High intensity interval training^a^	Training day/activity completed	Continuous moderate intensity training^b^
4 intervals	Day 1^c^	20 minutes
5 intervals	Day 2^d^	29 minutes
6 intervals	Day 3^e^	33 minutes
Home day, 6 intervals prescribed	Day 4^e^	Home day, 33 minutes prescribed
7 intervals	Day 5^e^	36 minutes
8 intervals	Day 6^e^	40 minutes
Home day, 8 intervals prescribed	Day 7^e^	Home day, 40 minutes prescribed
9 intervals	Day 8^e^	43 minutes
Home day, 9 intervals prescribed	Day 9^e^	Home day, 43 minutes prescribed
10 intervals	Day 10^c^	50 minutes

^a^One high intensity interval consists of 1 minute at ~80-90% VO_2_ peak and one minute ~40% VO_2_ peak.

^b^Moderate intensity continuous exercise consists of continuous exercise at ~45-55% VO_2_ peak.

^c^Stationary bike.

^d^Outdoor walking (hills for HIIT, flat for MICT).

^e^Participant choice (includes stationary bike, treadmill, elliptical, outdoor walking).

### High-Intensity Interval Training Protocol

HIIT involves sessions of 4 to 10 ×1-minute high-intensity intervals (cycling *sprints*, uphill treadmill walking, uphill outdoor walking, and elliptical machine) at approximately 80% to 90% peak oxygen uptake (VO_2_ peak), interspersed with 1-minute rest periods at approximately 40% VO_2_ peak. Results from the pilot study preceding this randomized controlled trial demonstrated that individuals with prediabetes respond well to this protocol [30].

### Moderate-Intensity Continuous Training Protocol

MICT involves sessions of 20 to 50 minutes continuous moderate-intensity exercise (cycling, treadmill walking, outdoor walking, and elliptical machine) at approximately 45% to 55% VO_2_ peak. This prescription of MICT matches the progression of HIIT (percent increase over time) and modeled the exercise program that has been shown to prevent the progression of prediabetes to T2D (ie, primarily walking; [11]). An intensity of approximately 45% to 55% VO_2_ peak, which approximates brisk walking [45,46], has been shown to improve cardiometabolic health in several large trials [47,48] and is commensurate with the level of intensity recommended by the American College of Sports Medicine [49] for inactive adults to promote exercise adherence.

### Transition to Independent Exercise

The 2-week supervised exercise training program will incorporate 3 unsupervised sessions to enable participants to practice engaging in exercise independently. Following completion of the 2-week program, participants will be asked to engage in their randomized exercise protocol (ie, HIIT or MICT) a minimum of 3 times per week for the next 12 months. Specifically, participants in HIIT will be prescribed a 5-minute warm-up, 10 ×1-minute high-intensity intervals, and a 5-minute cooldown. Participants in MICT will be prescribed 50 minutes of moderate-intensity exercise.

### Exercise Counseling

To assist participants with the transition to independent exercise and to promote long-term exercise adherence, participants in both conditions will receive a brief behavioral exercise counseling intervention built into the 2-week supervised training program. The counseling involves 10 minutes of one-on-one structured discussion at the end of each supervised exercise session, totaling 70 minutes over the 2 weeks. Participants will see the same counselor each session to bolster rapport. A detailed description of the BCTs used in the behavioral intervention and the social cognitive mechanisms they are hypothesized to target is reported elsewhere (Bourne et al, unpublished data). Participants will receive a worksheet for each of the 3 home-based sessions, each reviewing specific behavior change constructs.

In summary, the key aims of the behavioral exercise counseling are to:

1. Enhance confidence to perform the exercise (ie, task self-efficacy): providing instruction on how to perform the behavior, opportunities to practice engaging in exercise, helping participants identify physiological cues associated with the assigned exercise intensity, sharing the success stories of similar individuals, and providing positive support.
2. Increase confidence to self-manage exercise (ie, self-regulatory efficacy): providing opportunities to practice self-monitoring behavior, preplan exercise behavior, work through exercise barriers, and practice independent exercise.
3. Increase awareness of the psychological and physiological outcomes associated with exercise engagement (ie, outcome expectations): through education and drawing on the experiences of similar individuals and of the participant throughout the 2-week intervention.

The counseling will follow a semistructured format, targeting specific psychological and behavioral constructs each session, while allowing the participants to share their questions and concerns. The content of the program, tools, and materials to be used; session description; and the underlying theoretical constructs are presented in Multimedia Appendix 2.

### Mobile Phone–Based Self-Monitoring Application

To promote exercise adherence after the 2-week intervention, participants will be provided with a self-monitoring mobile application (app; [50]). The counselor will create the participant’s profile on the app and will show the participant how to navigate through the app on both a smartphone and a computer. Participants will be encouraged to monitor their exercise behavior daily (ie, record exercise into the app, hereon in referred to as *check-ins*) regardless of whether purposeful exercise was planned or completed that day. Participants will be permitted to record 4 nonexercise days within the app per week, coinciding with the prescribed 3 bouts of planned exercise for each week. Once all 4 *rest days* had been used, participants will record a *missed session* on days on which no exercise is completed.

Participants will be reminded to record their exercise behavior each day via an automated SMS text message sent from the app if they have not checked in by 9:00 pm. Participants will be informed that the counselor is able to view their exercise behavior through the counselor portal of the app. In the event of 3 consecutive missed check-ins, the participant will be contacted by the counselor via the app messaging function. During the 12-month follow-up period, the participant will be messaged monthly using the app messaging system on months with no scheduled follow-up appointments (months 1, 2, 4, 5, 7, 8, 10, and 11). These SMS text messages will target verbal persuasion, performance accomplishment, vicarious experience, awareness of physiological and affective cues, social support, self-monitoring, relapse prevention, and action planning.

### Outcomes and Measures

A timeline for when each measure was assessed is provided in Multimedia Appendix 1.

#### Primary Outcomes

The primary outcome is cardiorespiratory fitness determined by measuring VO_2_ peak at 6- and 12 months postrandomization. VO_2_ peak will be assessed by a continuous incremental ramp maximal exercise test on an electronically braked cycle ergometer (Lode Excalibur, The Netherlands). Expired gas will be collected continuously by a metabolic cart (Parvomedics TrueOne 2400, Salt Lake City, Utah, USA) that is calibrated with gases of known concentration and a 3.0 L syringe before every test. The test begins with a 4-minute warmup at 30 Watts, after which Watts increase by 1 every 4 seconds (15 Watts per minute). Verbal encouragement will be provided to the participant throughout the test. The test will be terminated upon volitional exhaustion or when revolutions per minute fall below 50. VO_2_ peak is defined as the highest 30-second average for VO_2_ (in L/min and mL/kg/min). Criteria for achieving VO_2_ peak are (1) respiratory exchange ratio >1.15; (2) plateau in VO_2_; (3) reaching age-predicted heart rate (HR) peak (220-age); and/or (4) volitional exhaustion. A Polar chest strap, which is integrated with the metabolic cart and cycle ergometer software (Lode Exercise Manager), will capture HR. HR peak and peak power output will be recorded as the highest values attained in the test. Cardiorespiratory fitness will be assessed at baseline and 6- and 12-month follow-up.

#### Secondary Outcomes

##### Physical Activity

Objectively measured purposeful MVPA adherence (ie, MVPA10+) will be assessed by triaxial accelerometry (Actigraph GT3X-BT, Actigraph, Pensacola, Florida, USA) at baseline and 3-, 6-, 9-, and 12-month follow-up. MVPA10+ is appropriate for measuring purposeful exercise [51] and provides a conservative estimate of exercise adherence. Participants will be instructed to wear the accelerometer on their right hip for 7 consecutive days at each measurement time point. Participants will be instructed to remove the accelerometer during sleep and for water-based activities. Accelerometers will be initialized and downloaded using ActiLife version 6.11. Epoch lengths will be specified at 5 seconds and will be summed as counts per minute. Nonwear time will be classified as 90 minutes of consecutive zeros, allowing for nonzero counts up to 2 minutes; if no counts are detected during the 30-minute counts before and after this interval [52], these data will be classified as nonwear time and excluded from analysis. Participants must have a total of ≥10 hours of valid wear time per day to be included in the analyses [53]. Our pilot work confirmed that recovery intervals during HIIT register as moderate-to-vigorous activity, not interruptions to physical activity. Freedson cut points [54] will be used to identify time spent in moderate (1952-5724 counts/min), vigorous (≥5725 counts/min), and MVPA (≥1952 counts/min) during wear time on valid wear days. Time spent in the various intensities will be averaged across valid wear days and multiplied by 7 to provide a weekly estimate of physical activity at each measurement time point. Purposeful exercise will be operationalized as minutes spent in MVPA10+ [54], in line with physical activity guidelines, which specify that bouts of physical activity should be accumulated in bouts of 10 minutes or more. Furthermore, given that participants in HIIT are prescribed 75 minutes of purposeful exercise per week (ie, 3×25-min HIIT sessions) and participants in MICT are prescribed 150 minutes per week (ie, 3×50-min MICT sessions) and that physical activity guidelines highlight the equivalency of 75 minutes of vigorous exercise and 150 minutes of moderate exercise, additional supplemental analyses will be conducted, whereby each vigorous minute of exercise within the identified MVPA10+ bouts will be credited as 2 minutes. This approach was carefully considered for the adherence measure and justified in the grant application for this trial. As such, this modified MVPA10+ outcome represents an appropriate measure of purposeful exercise [51] and allows for a direct assessment of adherence to HIIT or MICT.

Self-reported physical activity adherence will be assessed through an exercise log completed through the mobile application. Participants will be asked to record (1) whether exercise was complete, (2) type of exercise completed, (3) the duration of the exercise, (4) the number of intervals conducted (for the HIIT condition exclusively), and (5) how hard the session was (Rating of Perceived Exertion scale; [55]). The percentage adherence will be calculated (ie, number of exercise sessions divided by the number of prescribed sessions multiplied by 100% [56]).

##### Psychological Measures

Task self-efficacy, self-regulatory efficacy, concurrent self-regulatory efficacy, outcome expectations, and self-monitoring will be assessed at baseline, post intervention, and at 6- and 12-month follow-up (see Table 2 for specific details on study measures). Additional psychological variables that will be assessed include (1) exercise enjoyment measured at baseline, post intervention, and at 6- and 12-month follow-up [57]; (2) instrumental and affective attitudes measured at baseline, post intervention, and at 12-month follow-up (adapted from Conner et al [58]); and (3) affective state measured at baseline, post intervention, and at 12-month follow-up [59].

On training days 1, 6, and 10, counselors will ask participants to rate their in-task affect [60] and rating of perceived exertion [55] at the beginning (2.5%), middle (42.5%), and end (92.5%) of workout completion in both conditions (percentages indicate the proportion of workout completion to ensure this was matched across sessions of different lengths). In addition, participants will be asked to rate their exercise enjoyment halfway through the exercise session (50% of workout completion) in both conditions.

##### Anthropometric and Demographic Measures

Body mass, height (SECA, 700 SECA Hamburg Germany), waist circumference (measured at the level of the umbilicus), and blood pressure (measured manually using a sphygmomanometer) will be measured in the morning after an overnight fast at baseline, post intervention, and at 6- and 12-month follow-up using standard procedures. Information on ethnicity, household income, marital status, education level, medication history, and current smoking status will be collected at baseline.

##### Body Composition

Dual-energy x-ray absorptiometry (Hologic Discovery A) scans will be used to assess percentage body fat mass at baseline, post intervention, and at 12-month follow-up.

##### Biochemical Variables

A qualified and experienced phlebotomist will obtain a blood sample in the fasted state (≥8 hours overnight fast) and exactly 30 minutes following consumption of the 75 g glucose drink (Trutol, Fisher Scientific) from an antecubital vein. Samples will be placed on ice and processed within 30 minutes to obtain plasma via centrifugation for 15 minutes at 1550 g at 4℃.

**Table 2:** Description of psychological constructs assessed in the *Small Steps for Big Changes* program.

Outcome	Measure	Items	Exemplar question	Response options
Task self-efficacy	Study-specific. Created following recommendations made by Bandura [25] and McAuley and Mihalko [61]	4	How confident are you that you can perform 4-high intensity intervals OR perform 20-minutes of continuous moderate exercise (dependent on condition)	0% (not at all) to 100% (extremely confident)
Self-regulatory efficacy	Study-specific. Adapted from Shields and Brawley [62]	14	How confident are you that you can develop solutions to cope with time management challenges with respect to your exercise schedule?	0% (not at all) to 100% (extremely confident)
Concurrent self-regulatory efficacy	Study-specific. Adapted from Jung et al [19]	5	During a typical week, how confident are you in your ability to concurrently manage high-intensity interval training /continuous moderate intensity exercise amongst your other valued life goals? (dependent on condition)	0% (not at all) to 100% (extremely confident)
Outcome expectations: likelihood	Study-specific. Adapted from Locke et al [31]	23	How likely is it that each outcome in the list below will occur at least once in a typical week for the next four weeks as a result of engaging in high-intensity interval training/moderate intensity continuous training (dependent on condition) Lower risk of type 2 diabetes; Feel good about my physical appearance	1 (very unlikely) to 9 (very likely)
Outcome values	Study-specific. Adapted from Locke et al [31]	23	How much do you value attaining each outcome from the list below? Lower risk of type 2 diabetes; Feel good about my physical appearance	1 (little value to me) to 9 (great value to me)
Self-monitoring	Study-specific. Adapted from Hallam and Petosa [63] and Petosa [64]	19	In the past week I mentally kept track of my exercise activities	1 (never) to 5 (very often)
Affective and instrumental attitudes	Study-specific. Adapted from Connor et al [58]	13	For me, exercising three days per week would be...1 (useless) to 7 (useful)	1 to 7 (anchors vary depending on the question)
Affective state	The Positive and Negative Affect Schedule (PANAS; [59])	20	Read each item and then use the scale to indicate to what extent you have felt this way during the past few days (Interested, Distressed, Upset)	1 (very slightly or not at all) to 5 (extremely)
Enjoyment	Physical activity enjoyment scale (PACES; [57])	18	Please rate how you feel at the moment about the physical activity you have been doing...1 (I enjoy it) to 7 (I hate it)	1 to 7 (anchors vary depending on the question)
In-task affect	Feeling Scale [60]	1	When asked please tell me how you feel at that current moment using the scale below	+5 (very good) to −5 (very bad)
In-task rating of perceived exertion	10-point Category-Ratio Scale [55]	1	Using the scale provided please rate how hard you are currently working	0 (no exertion at all) to 10 (maximal exertion)
In-task exercise enjoyment	Study-specific	1	Using the scale provided please rate how much you are enjoying this exercise session	1 (note at all) to 7 (extremely)

Samples will be batch-analyzed in duplicate after storage at −80℃. Plasma glucose will be assessed via the enzymatic hexokinase method using a commercial assay (Pointe Scientific) on a clinical chemistry analyzer (Chemwell 2910, Awareness Technologies). Insulin will be measured by enzyme-linked immunosorbent assay (Mercodia, Upsula, Sweden), as described previously [65]. Glucose (mmoL/L) and insulin (mU/L) values will be used to calculate homeostasis model assessment of insulin resistance using the Web-based Homeostasis Model Assessment-2 calculator, a validated method that is highly correlated (*r*=.88) with the hyperinsulinemic-euglycemic clamp [66] and is sensitive to change following exercise interventions [65,67,68]. Oral glucose disposition index will be used to assess beta-cell function and will be calculated based on the hyperbolic relationship between glucose-stimulated insulin secretion (change in plasma insulin divided by the change in plasma glucose from fasting to 30 minutes) and insulin sensitivity (1/fasting insulin, as described by Utzschneider et al [68]). The inflammatory marker C-reactive protein will also be assessed. Postintervention samples will be obtained approximately 48 hours following the last supervised training session and at 12-month follow-up.

##### Continuous Glucose Monitor

CGM will take place using the iPro 2 Professional CGM and Enlite sensor (Medtronic MiniMed, Northridge, CA) in a subsample of participants. The first 20 participants randomized in the trial will be invited to participate in the CGM assessment. The CGM records glucose values in a blinded fashion using a small microneedle inserted into the subcutaneous abdominal adipose tissue that is connected to a recorder that quantifies interstitial glucose values every 5 minutes. Finger-stick blood glucose samples are taken 4 times per day and are analyzed by a software program (CareLink Pro) upon device removal and downloaded to create 24-hour blood glucose curves. CGM data will be analyzed for (1) average blood glucose, (2) 2-hour postprandial area under the glucose curve, (3) postmeal spikes, and (4) glycemic variability (mean amplitude of glycemic excursions and SD). Participants who wear a CGM will be provided a standardized diet (approximately 24 kcal/kg, approximately 60% carbohydrates, approximately 27% fat, and approximately 13% protein) to consume during the day before and on the day of each 24-hour monitoring period. CGM data will be collected for 24 hours at baseline and beginning approximately 24 hours after the last day of supervised training. To accurately interpret CGM data, each participant will consume the same meals at the same times of the day during each assessment period.

### Participant Safety

The primary safety concerns for participants in this trial will be associated with completing the cardiorespiratory fitness test and increased physical activity in daily life. These concerns include cardiovascular and musculoskeletal events. All participants will be cleared for exercise using the PAR-Q+ [43], and if required, their family physician will be asked to assess the participants’ readiness to engage in exercise and provide a completed ePARmed-X+ [43]. Participants that are not cleared for exercise by 1 of these 2 methods will be excluded from the study. During the cardiorespiratory fitness test, participant’s blood pressure and HR will be monitored and the test will be terminated if blood pressure exceeds 220/120 mmHg or an abnormal response is recorded. The supervised exercise period is designed to gradually introduce participants to exercise by increasing the amount of physical activity performed each day. In addition, participants have the autonomy to choose their preferred exercise format (eg, walking and elliptical) for 7 of the 10 training days, further reducing the risk of musculoskeletal discomfort. Injuries sustained because of exercise will be reported. All training and supervising staff are first aid trained.

### Intervention Fidelity

To promote the fidelity of intervention delivery and to ensure the underlying BCTs are administered as intended, a comprehensive 2-day counselor training workshop will be delivered by the principal investigator (an expert in BCTs). To augment the workshop, a standard operating procedure manual will guide intervention delivery for each day of the 2-week training program and for all follow-up face-to-face contact points (ie, 3-, 6-, 9-, and 12-months). The manual consists of detailed scripts and protocols for each exercise session, phone call, or app interaction. Counselors, who will be health psychology graduate students, will complete a checklist for each training day and follow-up session to ensure all key intervention components are administered. Checklists will align with information contained within the intervention scripts, for example, *reviewed and discussed barriers sheet completed by participant.* The 2-day training workshop covers theory and BCTs targeted in the intervention and is mandatory for all counselors. Each counselor will participate in role-play activities during the workshop that will be observed by the primary investigator, and feedback will be provided. Counselors will record intervention attendance, missed sessions, session time changes, or dropout for each participant throughout the intervention and follow-up.

During the follow-up phase, counselors will be provided with standardized monthly messages to send to each participant either using the app messaging system or via email in the months when no face-to-face contact was scheduled. Counselors will record the details of all contact with participants during this time.

### Statistical Procedures

#### Sample Size

The a priori sample size was derived in relation to the primary outcome for this trial, cardiorespiratory fitness assessed at 6 and 12 months. To calculate the sample size, a pooled mean of 20.8 mL/kg/min and a SD of 4.0 mL/kg/min were used based on our previous pilot study [30]. At the time of study design, the meta-analyses of Weston et al [69] reported that supervised HIIT led to approximately twice (approximately 19%) the improvement in VO_2_ peak as compared with MICT (approximately 10%) in participants with lifestyle-induced metabolic disease. We, therefore, used a 9% difference between HIIT and MICT as a meaningful exercise-induced increase in our population of interest, corresponding to a Cohen *d* effect size of 0.48 (or Cohen *f* of 0.24). Using these data, with a two-tailed alpha of .05 and 80% power, assuming a medium correlation among repeated measures of *r*=.5, it is calculated that 15 participants are required per group for a significant within-between interaction (calculated using G*Power v3.1; Department of Psychology, University of Duesseldorf).

However, given that the secondary outcome of exercise adherence typically produces greater measurement variability, the final sample size is determined to be powered for detecting a clinically relevant within-group increase in average daily MVPA assessed by an accelerometer. To calculate sample size, data from the Framingham Heart Study [51] and the Health Survey for England [70] were used. The Framingham data contain the most comprehensive cardiometabolic health measures matched with accelerometry-profiled physical activity in the world (N=2109, mean_age_=47 years, and female=54.53% (1150/2109). Using these data, an increase in 10 minutes of MVPA per day is associated with a 15% reduction in cardiometabolic risk [51]. An increase in approximately 9 min MVPA per day has been shown to reduce progression of prediabetes to T2D [71]. Thus, we calculated sample size to detect a 10-minute increase in average MVPA per day as this amount of activity is linked with improved cardiometabolic health and prevention of T2D. Given that in the Framingham study, the Actical rather than the Actigraph accelerometer was used, additional data were needed from which to derive the mean and SD for MVPA. To this end, the Health Survey for England data were used, which reported an association between physical activity and cardiometabolic risk factors, with MVPA mean values of 12 minutes per day (SD 16). This mean value does not differ markedly from other mean daily MVPA values in either the Canadian Health Measures Survey [72] or National Health and Nutrition Examination Survey [73]. To detect a difference of 10-minutes average MVPA per day within conditions, assuming a SD of 16, with 80% power at *P<*.05, 41 participants per group are required. A conservative loss to follow-up of approximately 20% is anticipated, and therefore, the trial aimed to recruit 50 participants per group (ie, 100 participants to be randomized).

#### Proposed Data Analyses

Preliminary analyses will be conducted to test for univariate and multivariate outliers and to test for normality. Data transformations will be conducted if required. Variance between the conditions in baseline outcome variables will be examined. Data will be analyzed on an intention-to-treat basis, with individuals retained within their randomized groups regardless of participation. All significance tests will be assessed at a 5% level of significance and effect sizes will be reported. Sensitivity analysis will be conducted to examine the impact of missing data. We will account for missing data by performing multiple imputation by chained equations to determine whether the results are impacted [74].

Mixed-effects regression with baseline as a covariate and 6- and 12-month outcomes as dependent variables will be used to examine differences in cardiorespiratory fitness between HIIT and MICT. Physical activity behavior assessed by accelerometer will be analyzed similarly but will include 3-, 6-, 9-, and 12-month time points. Mixed-effects regression using baseline and all other time points will be used to determine whether the treatments result in a significant change from baseline.

The mediating effects of task self-efficacy, self-regulatory efficacy, concurrent self-regulatory efficacy, outcome expectations, and self-monitoring will be examined using the INDIRECT macro developed by Preacher and Hayes [75]. The macro computes the following steps simultaneously: (1) regression coefficients for the impact of the intervention on the potential mediators; (2) the association between changes in the mediators and changes in the outcome variable; and (3) the total effects, direct effect, and indirect intervention effects. Bias-corrected bootstrapped 95% asymmetrical CIs will be computed for the indirect effect. Significant mediation will be established if the CIs do not include zero. Multiple mediation models will be computed for MVPA at 12-month follow-up and cardiorespiratory fitness at 12-month follow-up.

A series of mixed-effects regressions will be conducted to examine changes over time between groups in anthropometrics, body composition, biochemical markers, CGM, and additional psychological constructs.

### Data Management and Quality Assurance

The administrative database (ie, participant information) and questionnaire data will be managed in-house. Random checks will be performed on the entered survey data against paper records. All errors will be logged and corrected. All physiological, anthropometric, and biochemical measures will be conducted in 1 laboratory with established quality assurance systems. All data will be stored in a locked laboratory on password-protected and encrypted computers. Paper records will be stored in a locked filing cabinet.

### Retention

Maximizing retention is an important issue to fully understand exercise adherence rates over the 12-month follow-up. At recruitment, all laboratory visits and community visits will be outlined for the participants and the importance of follow-up testing emphasized regardless of exercise behavior. During the 2-week supervised intervention, participants will book the same appointment time for each day and will be sent all the dates and times by the counselor before day 1 of the intervention. Moreover, 2 of the follow-up appointments will be conducted within the local community at a location and time that suits the participant. It is anticipated that this will help reduce some of the burden on participants in attending a laboratory visit.

### Ethics Approval and Consent to Participate

Ethical approval was provided by the University of British Columbia Clinical Research Ethics committee (H12-02268-A008), with informed consent obtained from all participants in the study.

## Results

The project was funded in 2013 and enrollment began in July 2014. Data collection was complete in March 2017. Data analysis is currently underway, and the first results are expected to be submitted for publication in 2019.

## Discussion

The *Small Steps for Big Changes* program will be the first randomized trial to examine the efficacy of low-volume HIIT compared with traditionally prescribed MICT as a means of increasing long-term physical activity behavior in individuals at elevated risk of T2D. The impact of HIIT on cardiorespiratory fitness, physical activity adherence, and psychosocial outcomes will be directly compared with current exercise recommendations for this population (ie, moderate-intensity continuous exercise).

The authors acknowledge potential limitations in the proposed methodology. Specifically, exercise studies suffer from potential selection bias, which could impact the generalizability of the results. Furthermore, the considerable number of outcome measures may limit an individual’s willingness to participate. However, the number of outcome measures included in this study is similar to other exercise randomized controlled trials [76,77]. Generalizability of the findings may also be limited because of the study being conducted on a university campus, which may not reflect the environment of most individuals.

However, the findings from this study have the potential to make substantive contributions to understanding how different types of exercise protocol are tolerated and adhered to and the comparative health outcomes associated with these protocols. These findings could have significant implications for exercise recommendations provided for individuals at risk of diabetes.

In addition, this trial examines the effectiveness of a brief behavioral intervention designed to support the promotion of independent physical activity behavior through facilitation of self-regulatory strategies and efficacy beliefs. This intervention brings together theory and evidence in a carefully constructed intervention that will have the opportunity to examine the causal pathways from cognitions to exercise adherence. Together, the results of this trial have the potential to impact future public health campaigns designed to increase physical activity with individuals at risk of T2D.

## Appendix

Multimedia Appendix 1 Timeline for Small Steps for Big Changes schedule of enrolment, interventions, and assessments.

Multimedia Appendix 2 Small Steps for Big Changes intervention outline.

Multimedia Appendix 3 Peer-reviewer report from the CIHR.

## Abbreviations

BCT: behavior change technique
BMI: body mass index
CGM: continuous glucose monitoring
DPP: Diabetes Prevention Program
DPPOS: Diabetes Prevention Program Outcome Study
ePARmed-X+: Physical Activity Readiness Medical Examination
HIIT: high-intensity interval training
HR: heart rate
MICT: moderate-intensity continuous training
MVPA: moderate-to-vigorous physical activity
MVPA10+: moderate-to-vigorous physical activity in bouts of ≥10 min
PAR-Q+: Physical Activity Readiness Questionnaire for Everyone
SCT: social cognitive theory
SMS: short message service
T2D: type 2 diabetes
VO_2_peak: peak oxygen uptake
