# Supplementary figures and images for: Brief Exercise Counseling and High-Intensity Interval Training on Physical Activity Adherence and Cardiometabolic Health in Individuals at Risk of Type 2 Diabetes: Protocol for a Randomized Controlled Trial
Source: JMIR Res Protoc. 2019 Mar 26;8(3):e11226. doi: 10.2196/11226 (PMC6454331; doi:10.2196/11226)

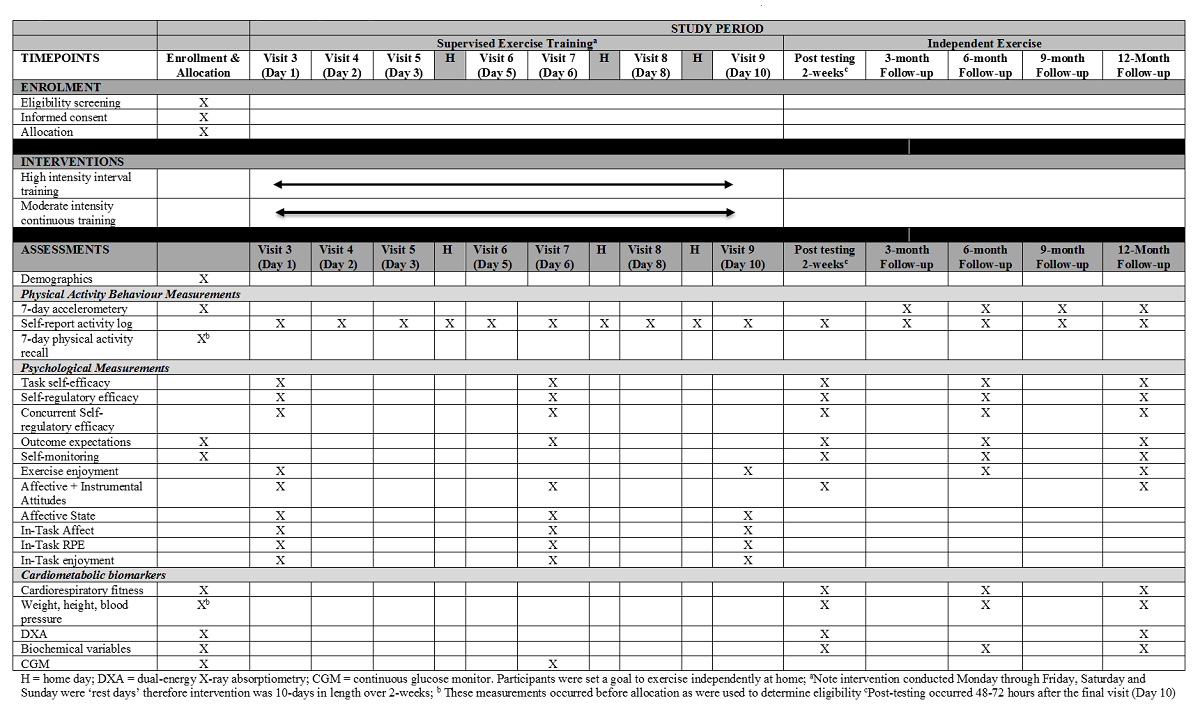

Supplement: Multimedia Appendix 1 [file resprot_v8i3e11226_app1.png]
