# Supplementary material for: Brief Exercise Counseling and High-Intensity Interval Training on Physical Activity Adherence and Cardiometabolic Health in Individuals at Risk of Type 2 Diabetes: Protocol for a Randomized Controlled Trial
Source: JMIR Res Protoc. 2019 Mar 26;8(3):e11226. doi: 10.2196/11226 (PMC6454331; doi:10.2196/11226)
Supplement: Multimedia Appendix 2 [file resprot_v8i3e11226_app2.pdf]

Appendix 1. Small Steps for Big Changes intervention outline.

Day	Main Aims of the Session	Tools	Targeted Constructs
**Day 1**[a]	Provide participant with a successful HIIT or MICT experience from which to build upon for the rest of the program through positive encouragement and support		Self-efficacy<br>• Mastery performance<br>• Verbal persuasion
	Show participant the Small Steps for Big Changes log book. Explain the importance of the logbook and encourage them to record their first exercise session. The participant should take control of bringing the logbook to each session and record his/her exercise	Exercise Log book	Self-monitoring of behaviour
**Day 2**[b]	Complete the exercise session outdoors to show the participant that he/she can complete the prescribed exercise without the use of equipment. Provide positive encouragement for completing exercise	Outdoor space	Self-efficacy<br>• Mastery performance<br>• Verbal persuasion<br>Social support
	Explain the physiological cues that the participant may experience during exercise and what these cues can tell the participant about the intensity of the exercise. Introduce the participant to the heart rate monitor that will help them match their target heart rate to the physiological cues that they experience	Heart rate monitor	Self-efficacy<br>• Physiological and affective cues
	Introduce the participant to the outcomes that he/she can expect to achieve from engaging in either HIIT or MICT. Discuss which outcomes the participant values from engaging in exercise		Outcome expectations<br>Outcome values
	Encourage the participant to complete log book	Exercise Log Book	Self-monitoring of behaviour
**Day 3**[c]	Let the participant choose what type of exercise he/she would like to engage in. Provide positive reinforcement throughout exercise session		Autonomy support<br>Self-regulatory efficacy<br>Self-efficacy<br>• Mastery performance<br>• Verbal persuasion
	Give positive reinforcement for completion of two days of exercise including one outdoor session without the need for equipment and one within the laboratory		Self-efficacy<br>• Mastery performance<br>• Verbal persuasion
	Encourage creation of a plan for the following day's		Action planning

	exercise at home. Provide positive encouragement for completing home exercise session		Self-efficacy<br>• Verbal persuasion
	Encourage participant to complete log book	Exercise Log Book	Self-monitoring of behaviour
**Day 4 Home Day**	Provide take home sheet to participants, including the following:<br>• Instructions on exercise duration and intensity to be completed<br>• Encourage participant to pay attention to the physiological cues they experience during exercise<br>• Use the heart rate monitor to ensure they are working at their target heart rate<br>• Match the physiological cues with their heart rate<br>• Complete logbook once exercise is complete<br>• Introduce the importance of rewards and acknowledgement of positive exercise behaviours<br>• Written positive encouragement for ability to complete the exercise	Take Home Sheet Day 4;<br>Heart rate monitor;<br>Exercise Logbook	Self-efficacy<br>• Master performance<br>• Physiological cues<br>•<br>Self-monitoring of behaviour<br>Self-regulatory efficacy<br>Positive reinforcement - reward
**Day 5** [c]	Give positive verbal feedback on past day's exercise behaviour and the participant's ability to complete exercise independently		Self-efficacy<br>• Verbal persuasion
	Discuss how the participant felt during and after the exercise session		Affect and affective attributions
	Let the participant choose what type of exercise they would like to engage in. Provide positive reinforcement throughout exercise session		Autonomy support<br>Self-regulatory efficacy<br>Self-efficacy<br>• Mastery performance<br>• Verbal persuasion
	Encourage participant to complete their log book	Exercise Log Book	Self-monitoring of behaviour
	Discuss the participants barriers to engaging in physical activity. Ask the participant to record these barriers in the sheet provided and to think about and record potential solutions that could help them overcome these barriers	Personal exercise barriers sheet	Relapse prevention
**Day 6** [c]	Draw attention to all the exercise the participant has completed in the first week of the program. Provide positive reinforcement for this activity		Self-efficacy<br>• Mastery performance<br>• Verbal persuasion
	Encourage the participant to use the physiological and affective cues to inform them about the exercise intensity	Heart rate monitor	Self-efficacy<br>• Physiological

	and to use the heart rate monitor to confirm exercise intensity		and affective cues
	Let the participant choose what type of exercise he/she would like to engage in. Provide positive reinforcement throughout exercise session		Self-regulatory efficacy<br>Self-efficacy<br>• Mastery performance<br>• Verbal persuasion<br>Autonomy support
	Encourage the participant to complete log book	Exercise Log Book	Self-monitoring of behaviour
	Recap on the outcomes the participant can expect to achieve from engaging in the prescribed exercise and discuss which outcomes the participant values the most		Outcome expectations<br>Outcome values
	Encourage creation of a plan for the follow day's exercise at home. Provide positive encouragement for completing home exercise session		Action planning<br>Self-efficacy<br>• Verbal persuasion
**Day 7 Home Day**	Provide take home sheet to the participant, including the following:<br>• Instructions on exercise duration and intensity to be completed<br>• Encourage participant to pay attention to the physiological cues they experience during exercise<br>• Use the heart rate monitor to ensure they are working at their target heart rate<br>• Match the physiological cues with their heart rate<br>• Complete logbook once exercise is complete<br>• Introduce the importance of reward and acknowledgement of positive exercise behaviours<br>• Encourage use of social and environmental support<br>• Written positive encouragement for ability to complete the exercise	Take Home Sheet Day 7;<br>Heart rate monitor;<br>Exercise Logbook	Self-efficacy<br>• Master performance<br>• Physiological cues<br>• Verbal persuasion<br>Self-monitoring of behaviour<br>Self-regulatory efficacy<br>Positive reinforcement – reward
**Day 8** [c]	Give positive verbal feedback on past day's exercise behaviour and the participant's ability to complete exercise independently		Self-efficacy<br>• Verbal persuasion
	Discuss how the participant felt during and after the exercise session		Affect and affective attributions
	Let the participant choose what type of exercise he/she would like to engage in. Ask the participant to monitor their exercise through their physiological and affective cues and using the heart rate monitor. Encourage participant to adapt intensity accordingly to reach the required intensity. Provide positive reinforcement	Heart rate monitor	Self-regulatory efficacy<br>Self-efficacy<br>• Mastery performance<br>• Verbal

	throughout exercise session		persuasion<br>Autonomy support
	Show the participant the Small Steps for Big Change video of past participants engaging in the same exercise prescription and the Small Steps 'rock star' board (images of individuals from previous study who were able to change their physical activity behaviour). Focus on the similarities between these individuals and the participant	Small Steps for Big Changes Stars Video; Small Steps Rock Star Board	Self-efficacy<br>• Vicarious experiences
	Reinforce the participant as a role model to other inactive individuals		Self-efficacy<br>• Verbal persuasion
	Encourage the participant to complete their log book	Exercise Log Book	Self-monitoring of behaviour
	Encourage creation of a plan for the follow day's exercise at home. Provide positive encouragement for completing home exercise session		Action planning<br>Self-efficacy<br>• Verbal persuasion
**Day 9 Home Day**	Provide take home sheet to the participant, including the following:<br>• Instructions on exercise duration and intensity to be completed<br>• Encourage participant to pay attention to the physiological cues they experience during exercise<br>• Use the heart rate monitor to ensure they are working at their target heart rate<br>• Match the physiological cues with their heart rate<br>• Complete logbook once exercise is complete<br>• Written positive encouragement for ability to complete the exercise<br>• Encourage participant to think about their exercise journey over the past few days and how much independent exercise they have completed	Take Home Sheet Day 9; Heart rate monitor; Exercise Logbook	Self-efficacy<br>• Master performance<br>• Physiological cues<br>• Verbal persuasion<br>Self-monitoring of behaviour<br>Self-regulatory efficacy
**Day 10**[a]	Give positive verbal feedback on past day's exercise behaviour and the participant's ability to complete exercise independently. Recap on all the exercise the participant has completed over the past 2-weeks and provide positive reinforcement for their ability to complete independent exercise moving forward		Self-efficacy<br>• Verbal persuasion<br>• Mastery performance
	Ask the participant to monitor their exercise through their physiological and affective cues. Encourage participant to adapt intensity accordingly to reach the required intensity. Provide positive reinforcement throughout exercise session		Self-regulatory efficacy<br>Self-efficacy<br>• Verbal persuasion

			• Physiological and affective cues
	Discuss with the participant that barriers will occur but they have created solutions on how to overcome these barriers should they arise		Relapse prevention
	Encourage the participant to complete his/her logbook. Review log book and provide verbal feedback on this behaviour	Exercise Log Book	Self-monitoring of behaviour<br>Positive reinforcement<br>Self-efficacy<br>• Verbal persuasion
	Congratulate the participant on completing two-weeks of exercise training and increasing exercise duration during that time. Remind participants that they have the knowledge, skills and strategies of complete independent exercise moving forward	Certificate of completion	Social support<br>Self-efficacy<br>Self-regulatory efficacy
	Introduce the participant to the online exercise tracking system	Online application;	Self-monitoring of behaviour
	Provide follow up booklet to the participant, including the following:<br>• Instructions on exercise duration and intensity to be completed<br>• Encourage participant to pay attention to the physiological cues they experience during exercise as an indicator of exercise intensity<br>• Complete online tracking tool once exercise is complete<br>• Remind participant of skills and knowledge they have obtained over the two-weeks to enable them to engage in independent exercise.<br>• Encourage participant to seek social and environmental support for exercise<br>• Remind participant that they have the strategies to overcome any barriers they face<br>• Remind the participant what outcomes they can expect to achieve from engaging in exercise	Follow-up Small Steps for Big Changes Book	Self-efficacy<br>• Mastery performance<br>• Physiological and affective cues<br>• Verbal persuasion<br>Self-monitoring of behaviour<br>Self-regulatory efficacy<br>Outcome expectations

Exercise format: [a]Stationary bike; [b]Outdoor walking (hills for HIIT, flat for MICT); [c]Participant choice (includes stationary bike, treadmill, elliptical, outdoor walking)
