# Supplementary material for: Brief Exercise Counseling and High-Intensity Interval Training on Physical Activity Adherence and Cardiometabolic Health in Individuals at Risk of Type 2 Diabetes: Protocol for a Randomized Controlled Trial
Source: JMIR Res Protoc. 2019 Mar 26;8(3):e11226. doi: 10.2196/11226 (PMC6454331; doi:10.2196/11226)
Supplement: Multimedia Appendix 3 [file resprot_v8i3e11226_app3.pdf]

Canadian Institutes of Health Research/Instituts de recherche en santé du Canada

Notice of Recommendation/Avis de recommandation

Application Number/Numéro de la demande: 312944

Committee Code/Code du comité: PB2

Applicants/Candidats: Dr. Mary Elizabeth JUNG   Dr. Jonathan Peter LITTLE

With/Avec: Dr. M. BEAUCHAMP   Dr. C. JONES   Dr. J. SINGER

Institution paid/ Établissement payé: University of British Columbia

Title/Titre: Integrating a brief, social cognitive theory-based intervention with high-intensity interval training to improve exercise adherence

Primary Inst./Inst. principal: Nutrition, Metabolism and Diabetes

Other Related Inst./ Autres inst. connexes: Musculoskeletal Health and Arthritis; Population and Public Health

Competition /Concours: Operating Grant
September/Septembre 16, 2013

Number in competition/Nbre de demandes dans le concours: 2528

Peer Review Committee Recommendation, for your information and use/
Recommandation du comité d'examen par les pairs, pour fins d'information et d'utilisation:

**Committee/Comité:**	Psychosocial, Sociocultural & Behavioural Determinants of Health 2
**Number reviewed/ Demandes examinées:**	44
**Application rank within the committee/ Rang de la demande dans le comité:**	7
**Percent Rank within the committee / Rang en pourcentage au sein du comité:**	15.91%
**Rated / Cote:**	3.96
**Recommended Term/ Durée recommandée:**	3 years/ans 0 months/mois
**Recommended average annual operating amount/ Montant annuel moyen recommandé pour le fonctionnement:**	$125,923
**Recommended equipment amount/ Montant recommandé pour les appareils:**	$0

This document is for information only.
An application rated below 3.50 is ineligible for CIHR funding. For applications rated 3.50 and above, please note that it is the application's rank within the peer review committee that determines whether it is funded, rather than its absolute rating. The final funding decision will be communicated in the Notice of Decision.

Document à titre d'information seulement.
Une demande cotée en dessous de 3,5 n'est pas admissible au financement des IRSC. En ce qui a trait aux demandes cotées 3,50 ou plus, veuillez noter que l'on détermine l'attribution des fonds en fonction du classement obtenu au sein du comité d'examen par les pairs plutôt qu'en fonction du classement absolu. La décision finale relative au financement sera communiquée dans l'Avis de décision.

Review Type/Type dévaluation: Committee Member 1/Membre de comité 1
Name of Applicant/Nom du chercheur: JUNG, Mary Elizabeth
Application No./Numéro de demande: 312944
Agency/Agence: CIHR/IRSC
Competition/Concours: 2013-09-16 Operating Grant/Subvention de fonctionnement
Committee/Comité: Psychosocial, Sociocultural & Behavioural Determinants of Health 2/Déterminants psychosociaux, socio-culturels et comportementaux de la santé 2
Title/Titre: Integrating a brief, social cognitive theory-based intervention with high-intensity interval training to improve exercise adherence

---

Assessment/Évaluation:

Synopsis

The primary aim of this RCT is to determine whether short-term, time-efficient, high-intensity interval training (HIT) based on social-cognitive therapy (SCT) leads to greater exercise adherence over one year than an SCT-based intervention using standard care continuous moderate-intensity exercise (MOD) in those with prediabetes. The secondary objective is to determine if HIT leads to greater improvements in cardiometabolic health when compared to MOD after two weeks of supervised training, and one year of free-living exercise; and a third objective is to examine the potential mediating role of SCT constructs on exercise adherence.

It is a three-year, single-centre, parallel-group design with 100 inactive adults with prediabetes. The two-week SCT intervention involves exercise counseling on goal setting, overcoming barriers, planning, and self-monitoring, coupled with supervised exercise training in the laboratory that gradually weans into independent home-based exercise. Participants will progress from one independent at-home exercise session in week 1 to two on their own in week 2. Post-intervention, participants will be prescribed to maintain, on their own, training of three sessions per week of either HIT or MOD for one year. To objectively monitor exercise adherence, accelerometers with integrated HR monitors will be used. Accelerometers will be worn for 7 day periods once per month for 12 months follow-up. Participants will be instructed to put the integrated HR monitor chest strap on when they perform purposeful activity during these 7 day periods in order to provide a digital time-stamped marker of exercise adherence. The primary outcome is time spent in moderate-to-vigorous physical activity (MVPA) during this purposeful activity over months 7-12 of follow-up. Secondary outcomes will include: (1) cardiorespiratory fitness (VO2peak) assessed at 2 weeks and 6 and 12 months; (2) purposeful MVPA over months 1-6 follow-up; (3) self-report percent adherence measured from monthly logs over months 1-12; and (4) blood markers of T2D and CVD risk.

All participants will be screened, including a 12-lead EKG, and stratified by age and sex.

A small feasibility trial showed that SCT training increased self-efficacy and that HIT is superior to MOD for improving cardiorespiratory fitness and is preferred over MOD.

Research Team

Dr. Mary Jung has a Ph.D. in Health and Exercise Psychology from the University of Saskatchewan, completed in 2008. She is currently an Assistant Professor in the Department of Human Kinetics at UBC. She is the PI on 1 grant from Medtronic, 2 from UBC, 1 from the Hampton Research Endowment Fund, and 1

**Review Type/Type dévaluation:**	Committee Member 1/Membre de comité 1
**Name of Applicant/Nom du chercheur:**	JUNG, Mary Elizabeth
**Application No./Numéro de demande:**	312944
**Agency/Agence:**	CIHR/IRSC
**Competition/Concours:**	2013-09-16 Operating Grant/Subvention de fonctionnement
**Committee/Comité:**	Psychosocial, Sociocultural & Behavioural Determinants of Health 2/Déterminants psychosociaux, socio-culturels et comportementaux de la santé 2
**Title/Titre:**	Integrating a brief, social cognitive theory-based intervention with high-intensity interval training to improve exercise adherence

Assessment/Évaluation:

from Dairy Farmers of Canada; and is a co-I on 4 others (none from CIHR). She had a CIHR PDF. She has 7 first-author publications, and is a co-author on 21 others; a solid record for a young researcher. The other members of the team bring expertise in kinesiology, epidemiology and biostatistics, endocrinology, and exercise psychology – a very strong team.

Critique
Overall, this is a very well thought out and well written proposal. My major concern is that of compliance; not so much with the program itself as with the requirement to wear the cardiac monitor for one week every month over a one year period. Unfortunately, there are no pilot data for this. It may be possible to mitigate this problem with telephone or email reminders, or to make the major outcome VO2peak. There is also no description of how they will examine their third aim, of looking at the mediating role of SCT constructs on adherence.

Budget
No concerns.

Review Type/Type dévaluation: Committee Member 2/Membre de comité 2
Name of Applicant/Nom du chercheur: JUNG, Mary Elizabeth
Application No./Numéro de demande: 312944
Agency/Agence: CIHR/IRSC
Competition/Concours: 2013-09-16 Operating Grant/Subvention de fonctionnement
Committee/Comité: Psychosocial, Sociocultural & Behavioural Determinants of Health 2/Déterminants psychosociaux, socio-culturels et comportementaux de la santé 2
Title/Titre: Integrating a brief, social cognitive theory-based intervention with high-intensity interval training to improve exercise adherence

---

Assessment/Évaluation:

Name of Applicant: JUNG, Mary

Title of project: Integrating a brief, social cognitive theory-based intervention with high-intensity interval training to improve exercise adherence

Reviewer: 2

Summary of project: Prediabetes is a metabolic condition in which individuals have abnormal glucose metabolism and are at high risk of progression to frank diabetes mellitus over 1-5 years. Diabetes mellitus is associated with significant morbidity, mortality, and costs. Exercise-based interventions have been proven in high-quality trials to reduce the progression to diabetes. However, adherence to exercise-based programs is poor among those with prediabetes. High-intensity interval training (HIT) is an alternative to conventional exercise programming and may be equally, if not more, effective as well as being associated with better adherence in pilot studies. The investigators propose an innovative study comparing HIT and a Social Cognitive Theory (SCT)-based intervention with a SCT-based intervention combined with conventional exercise programming (moderate intensity continuous exercise) in a randomized trial. The primary outcome is moderate to vigorous physical activity (MVPA). The sample size is 100 adults with prediabetes defined using standard criteria.

Critique:

This is an initial submission.

The scientific summary is fairly clear although the analytic approach is not specified and the change in the primary outcome that is being sought is not clear.

I found the lay summary to be written in language that is technical and above a grade 8 reading level (e.g. ‘adherence’, ‘dissemination’).

The summary of progress is clear and helpful. I was surprised that no senior investigator was mentioned in the initial work that has been done. Even though n=13 in the original feasibility study, I would have liked to see explicit mention of the effect size that was seen (recognizing it will obviously not be statistically significant given the sample size, but at least a reasonable indication of the possible magnitude of effect of the intervention).

I liked the synopsis. The aims were clear.

**Review Type/Type dévaluation:**	Committee Member 2/Membre de comité 2
**Name of Applicant/Nom du chercheur:**	JUNG, Mary Elizabeth
**Application No./Numéro de demande:**	312944
**Agency/Agence:**	CIHR/IRSC
**Competition/Concours:**	2013-09-16 Operating Grant/Subvention de fonctionnement
**Committee/Comité:**	Psychosocial, Sociocultural & Behavioural Determinants of Health 2/Déterminants psychosociaux, socio-culturels et comportementaux de la santé 2
**Title/Titre:**	Integrating a brief, social cognitive theory-based intervention with high-intensity interval training to improve exercise adherence

Assessment/Évaluation:

The study background is reasonable and generally detailed, although I found section 1.1.3, wherein brief SCT-based interventions are described, to be a bit vague and not sufficiently precise with quantitative summaries of results. Pilot data were nicely presented in section 1.1.5, although the clinical significance of the observed percentage change in VO2 peak was not specified and would have been helpful.

The need for a trial at present and risks of the HIT intervention were nicely laid out.

In terms of inclusion/exclusion criteria, most are reasonable. It is unclear why an age cut-off of 65 years was used, however, particularly with maximal exercise stress testing being done during screening. In addition, why was a BMI >40 an exclusion criterion if the patient is otherwise able to exercise safely? While this is likely reasonable, I would have liked to see a brief justification.

Once-monthly accelerometry for a year is ambitious, and I worry about participant burden and feasibility. This makes me wonder whether a slightly smaller study, with n=60, and a shorter time horizon of 6 months follow-up, might not be a more logical next step, given the pilot/feasibility data presented to date.

I liked Figure 3 as a nice way of summarizing multiple issues and concepts. However, I was worried that there were only 3 points of contact between 2 weeks and 12 months, risking significant attrition during ‘silent’ periods among participants. It may particularly favour the HIT group as well. I think the investigators should rethink this, particularly in an early phase trial such as the proposed.

Secondary and tertiary outcomes all seem reasonable. It is unclear to me what exercise logs will add to accelerometry data, particularly since the latter are being done monthly. Exercise logs are not terribly reliable and they are quite burdensome for participants. Better justification is needed for this.

I would have liked to see a little more detail in the description of the SCT construct measures in section 2.9.

While collecting QOL data using the SF-36 is reasonable, I disagree with the authors that the sample size of 100 will be enough to detect differences. Within-group differences over time may well be detectable, but between-group differences are unlikely to be seen with n=100, at least in the absence of additional data. However, this is a minor point since QOL is neither a primary nor major secondary outcome at this phase.

I found virtually no information on collecting adverse event information. Given the nature of the population being recruited and the HIT intervention, this is particularly important.

I found the sample size justification to be reasonable for the primary outcome. I would have liked to see data in the appendices to back up the statement that n=100 will be adequate for secondary outcomes. However, it

Review Type/Type dévaluation: Committee Member 2/Membre de comité 2
Name of Applicant/Nom du chercheur: JUNG, Mary Elizabeth
Application No./Numéro de demande: 312944
Agency/Agence: CIHR/IRSC
Competition/Concours: 2013-09-16 Operating Grant/Subvention de fonctionnement
Committee/Comité: Psychosocial, Sociocultural & Behavioural Determinants of Health 2/Déterminants psychosociaux, socio-culturels et comportementaux de la santé 2
Title/Titre: Integrating a brief, social cognitive theory-based intervention with high-intensity interval training to improve exercise adherence

Assessment/Évaluation:

is unclear to me why 12 months of follow-up is needed at this phase of the research program.

The recruitment approach seems reasonable.

The statistical analysis section was clearly written and sensible. I would have liked to see more details on data capture and handling.

I completely agree with having a DSMB established, although no details are provided on its make-up, frequency of meeting, and criteria for early study termination.

The timeline is detailed but suggests the study will start in January 2014, which is earlier than the funding decision date. This is probably a simple error.

The KT plan is reasonable.

A strong support letter was included from Dr. Esliger, who is clearly an international expert in his field. Multiple other support letters were included which strengthened the proposal, including a strong letter of support from the Canadian Diabetes Association.

Investigators: The PI and co-PI are relatively junior investigators at the University of British Columbia. They have collaborated in the past and bring together complementary clinical and lab expertise. The team is strengthened by a senior exercise trialist and methodologist.

Environment: No concerns, although the multiple statements about the topography of Kelowna are a bit hyperbolic.

Budget: Generally well-detailed and adequately justified.

Although I have no problem with the budget request for a probable PhD student, it would be nice to see whether involvement in the current project will be linked to his/her thesis and a possible thesis topic or area.

No data are presented on the inter-individual variability in accelerometer outputs to support the need for a dedicated accelerometer per participant. Nor is this mentioned in the specifications of the equipment, nor in the letter by Dr. Esliger. This is also unusual in clinical trials in this area.

There is no need to perform glucose or lipid measurements in duplicate. These are routinely performed with very limited coefficients of variability. Insulin and CRP are reasonable to perform in duplicate.

**Review Type/Type dévaluation:**	Committee Member 2/Membre de comité 2
**Name of Applicant/Nom du chercheur:**	JUNG, Mary Elizabeth
**Application No./Numéro de demande:**	312944
**Agency/Agence:**	CIHR/IRSC
**Competition/Concours:**	2013-09-16 Operating Grant/Subvention de fonctionnement
**Committee/Comité:**	Psychosocial, Sociocultural & Behavioural Determinants of Health 2/Déterminants psychosociaux, socio-culturels et comportementaux de la santé 2
**Title/Titre:**	Integrating a brief, social cognitive theory-based intervention with high-intensity interval training to improve exercise adherence

Assessment/Évaluation:

Other costs including travel appear reasonable.
